# Contemporary Area‐Level Mortgage Loan Denial Risk and Health‐Related Quality of Life Among Cancer Survivors

**DOI:** 10.1002/cam4.71433

**Published:** 2025-12-17

**Authors:** Jordyn A. Brown, Taylor D. Ellington, Leah Moubadder, Maya Bliss, Anjali D. Kumar, Chantel L. Martin, Laura Farnan, Adrian Gerstel, Lauren E. McCullough, Hazel B. Nichols

**Affiliations:** ^1^ Department of Epidemiology, Gillings School of Global Public Health University of North Carolina Chapel Hill North Carolina USA; ^2^ Department of Epidemiology, Rollins School of Public Health Emory University Atlanta Georgia USA; ^3^ Department of Environmental Health, Rollins School of Public Health Emory University Atlanta Georgia USA; ^4^ Carolina Population Center University of North Carolina Chapel Hill North Carolina USA; ^5^ Lineberger Comprehensive Cancer Center University of North Carolina Chapel Hill North Carolina USA

**Keywords:** cancer survivorship, quality of life, residential segregation, social determinants of health, structural inequities, structural racism

## Abstract

**Background:**

Historical and contemporary mortgage lending practices have been associated with worse cancer outcomes. We estimated the association between mortgage loan denial risk and health‐related quality of life (HRQoL) among survivors at a large tertiary medical center in North Carolina (NC).

**Methods:**

Mortgage denial risk, calculated using Home Mortgage Disclosure Act data (2010–2014), was linked by census tract to UNC Cancer Survivorship Cohort survivors who resided within NC metropolitan statistical areas (MSAs). HRQoL was measured using the validated Functional Assessment of Cancer Therapy‐General (FACT‐G). Differences in well‐being across seven MSAs were assessed using Pearson chi‐square tests and modified Poisson regression models adjusted for demographic and socioeconomic characteristics.

**Results:**

Over 40% of survivors reported low overall well‐being, while more than one‐third of survivors reported low physical, social, emotional, or functional well‐being. FACT‐G scores were 6.2 (95% CI: 1.5, 10.9) points lower among Greensboro survivors; 4.1 (95% CI: 0.1, 8.1) points lower among Fayetteville MSA survivors; and 9.6 (95% CI: 2.5, 16.8) points lower among Charlotte survivors at a higher risk for mortgage loan denial compared to those at lower risk. Racial differences in FACT‐G scores were only observed among Greensboro and Charlotte MSA survivors, whereas sex differences were limited to Charlotte survivors.

**Conclusions:**

Mortgage loan denial was associated with worse overall HRQoL in Greensboro, Fayetteville, and Charlotte, but not in other NC MSAs. Further investigation into the role of place in other NC MSAs is needed to identify opportunities to support survivors across the cancer control continuum.

## Graphical abstract

Mortgage loan denial risk is associated with significantly lower health‐related quality of life among cancer survivors in some metropolitan statistical areas of North Carolina. Our findings highlight the need to explore place‐based factors to better support survivor well‐being across the cancer control continuum.

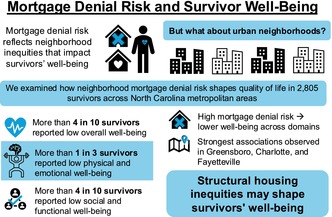

## 1 Introduction

Cancer is the second‐leading cause of death in North Carolina and the U.S. [1, 2], with growing recognition that the social determinants of health, in particular housing conditions and neighborhood resources, are shaped by the policies, practices, and resources that contribute to disparate cancer outcomes [3, 4, 5]. Urban cancer survivors generally have better access to healthcare and cancer recovery opportunities compared to rural survivors [6, 7, 8, 9, 10]. However, limited research examines how inequitable social structures within urban areas, especially structural housing factors, affect survivorship outcomes [11, 12, 13].

Neighborhood‐level inequities represent modifiable upstream structural determinants of health that influence survivor well‐being throughout the cancer care continuum [14, 15, 16, 17]. Housing represents a critical, yet understudied, structural determinant of health. Discriminatory practices, such as biased mortgage lending and rental discrimination, have historically limited homeownership and economic mobility among underserved groups, disproportionately affecting underrepresented racial and ethnic populations [14, 15, 16, 18, 19]. These patterns reinforce racialized and economically segregated neighborhoods with limited health‐promoting resources and greater exposure to poor nutrition, transportation, and healthcare [18, 19, 20, 21].

Such neighborhood conditions can directly impact health‐related quality of life (HRQoL), a multidimensional, patient‐reported outcome strongly associated with other clinical cancer outcomes [22, 23, 24, 25, 26]. HRQoL is influenced by access to treatment [27], social support [28], and geographic context, including urban–rural differences [29, 30, 31, 32, 33]. However, few studies examine how structural housing inequities in urban areas affect survivors' HRQoL.

Our study investigates mortgage loan denial risk, a proxy for systemic barriers to homeownership, as an indicator of broader social disadvantage affecting survivorship. Underserved racial and ethnic groups face higher denial rates and often reside in neighborhoods with fewer health‐promoting resources [14, 15, 16, 18, 19, 34, 35]. Using a metropolitan statistical area (MSA)‐specific mortgage denial risk measure, we focus on urban survivors across North Carolina to assess how structural housing inequities affect HRQoL. We examine the association between mortgage loan denial risk and HRQoL to highlight how structural housing inequities may shape survivor outcomes.

## 2 Materials and Methods

### 2.1 Study Design and Population

The University of North Carolina (UNC) Cancer Survivorship Cohort (CSC) is a large, tertiary medical center‐based cohort of adult cancers that has been previously described [36]. Between April 2010 and August 2016, English‐ or Spanish‐speaking patients aged 18 years or older were recruited from an outpatient oncology clinic. Among consenting participants, 3999 with a UNC Tumor Registry‐confirmed cancer completed a 60‐min baseline questionnaire in person or via telephone within 2 weeks of enrollment. Participants responded to general health and cancer‐specific items, such as individual sociodemographics, health history, lifestyle, and HRQoL indicators.

For this analysis, we excluded participants with missing HRQoL (*n* = 15), race (*n* = 32), marital status (*n* = 23), and education or employment (*n* = 18). We further excluded 264 participants with multiple cancer diagnoses, whose medically complex care needs and experiences may differ from those with a single primary cancer. We also excluded 842 participants living outside MSAs because our exposure was derived only for North Carolina MSA populations, yielding a final analytic sample of 2805 survivors. This project was reviewed and approved by the Human Research Protections Program (IRB Number: 23‐1585) at the University of North Carolina at Chapel Hill.

### 2.2 Exposure Assessment

**FIGURE 1:**
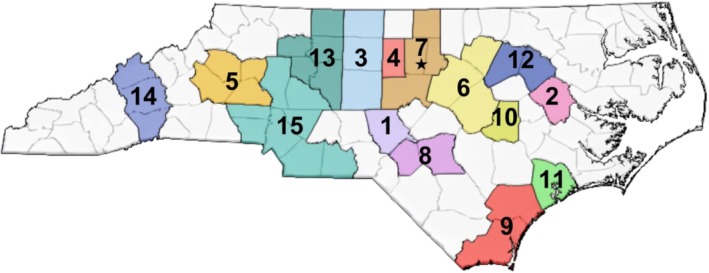
Map of metropolitan statistical areas across North Carolina. The metropolitan areas in North Carolina are as follows: (1) Pinehurst‐Southern Pines (formerly known as New Bern); (2) Greenville; (3) Greensboro‐High Point; (4) Burlington; (5) Hickory‐Morganton‐Lenoir; (6) Raleigh; (7) Durham‐Chapel Hill; (8) Fayetteville; (9) Wilmington; (10) Goldsboro; (11) Jacksonville; (12) Rocky Mount; (13) Winston‐Salem; (14) Asheville; and (15) Charlotte‐Concord‐Gastonia. The star marks the main study site location where recruitment occurred at the UNC Lineberger Comprehensive Center oncology outpatient clinic.

We used a publicly available measure of area‐level mortgage denial risk developed by Moubadder and colleagues [37], using data from the Home Mortgage Disclosure Act (HMDA). Briefly, the HMDA, enacted by Congress in 1975, requires financial institutions to publicly disclose mortgage lending data, including applicant characteristics, such as race, ethnicity, sex, and income, in addition to loan characteristics and census tract level information [38, 39, 40]. Participants' home addresses at baseline were geocoded and linked to HMDA data on mortgage applications for MSAs, which consist of urbanized areas with ≥ 50,000 residents [41]. MSAs were chosen to account for a common mortgage lending market with similar economic and social policies. Among UNC CSC participants, we identified 1738 census tracts nested within 15 North Carolina MSAs (Figure 1).

Mortgage loan denial risk was estimated using a hierarchical Bayesian spatial model developed by Moubadder and colleagues [37], which calculates the relative risk of mortgage loan denial for each census tract compared to the average loan denial risk of the MSA as a whole (all census tracts combined), adjusting for applicant sex and a ratio of loan amount to applicant gross annual income. The measure was based on loan‐level information from 2010 to 2014 HMDA data: mortgage loan outcomes (e.g., approved, denied, withdrawn); loan type (e.g., conventional, Veterans Administration–guaranteed, or Federal Housing Administration–insured, or Farm Service Agency or Rural Housing Service); loan purpose (e.g., home purchase, home improvement, refinancing); applicant sex; and the census tract of the property for which the loan was applied. A relative risk > 1 indicates a higher risk of mortgage denial than the MSA average, whereas a relative risk ≤ 1 indicates lower risk.

This mortgage loan denial risk measure was intentionally designed to address key limitations of previous approaches to quantifying mortgage denial risk. Specifically, Moubadder and colleagues accounted for spatial autocorrelation between census tracts, producing locally stable and reproducible estimates of lending risk. Importantly, the model does not adjust for race, recognizing the systemic and structural nature of racialized economic exclusion. By explicitly modeling spatial clustering, the measure identifies neighborhoods experiencing disproportionate mortgage denial risk relative to the broader MSA and thereby captures localized inequities that contribute to neighborhood disinvestment and related health inequities. Further methodological detail about the mortgage denial measure is described elsewhere [37].

We adapted the measure developed by Moubadder and colleagues to facilitate cross‐MSA comparisons while preserving local variation. Within each MSA, we standardized tract‐level values and dichotomized census tracts using both unstandardized and standardized measures. For the unstandardized measure, tracts were categorized as higher (> 1) or lower (≤ 1) risk relative to the MSA average. For the standardized measure, tracts were categorized as higher (> 0) or lower (≤ 0) risk relative to the MSA average. Dichotomizing both measures simplified interpretation and aligned with prior research on area‐level lending inequities. Although dichotomization may obscure finer gradients, limited participants per MSA prevented further sensitivity analyses.

### 2.3 Outcome Assessment

The Functional Assessment of Cancer Therapy‐General (FACT‐G) is a 27‐item questionnaire that assesses HRQoL across four domains: physical (seven items), social (seven items), emotional (six items), and functional well‐being (seven items) [42]. Participants rate each item on a 5‐item Likert scale (0 = “not at all” to 4 = “very much”) based on the past 7 days, with higher scores indicating better HRQoL. Scores were dichotomized using validated cancer population norms [43]: overall FACT‐G (range 0–108) at 80.9; physical (range 0–28) at 21.3; social (range 0–28) at 22.1; emotional (range 0–24) at 18.7; and functional (range 0–28) at 18.9. Clinically meaningful differences correspond to a 5‐point difference on overall FACT‐G scores and 2‐point differences on subscale scores, consistent with prior studies [44, 45, 46].

### 2.4 Statistical Analysis

We calculated univariate descriptive statistics, marginal mean FACT‐G scores, and 95% confidence intervals (CIs) with robust standard errors by survivors' demographic and cancer‐related characteristics. Pearson chi‐square tests assessed differences in low HRQoL prevalence by mortgage denial risk group. FACT‐G scores between high and low mortgage denial risk groups were compared using generalized linear models with a Poisson distribution, identity link, and robust standard errors, appropriate for modeling common binary outcomes [47]. Analyses were restricted to MSA residents, with overall associations across all 15 MSAs (Figure 1) and MSA‐specific analyses for seven MSAs (Greensboro‐High Point, Raleigh, Durham‐Chapel Hill, Fayetteville, Wilmington, Rocky Mount, and Charlotte‐Concord‐Gastonia) with ≥ 21 participants. To account for contextual heterogeneity, we used linear mixed‐effect models with MSA‐specific random intercepts and slopes for standardized mortgage denial risk groups.

Potential confounders were selected based on a scientific literature review [48, 49] and included age at diagnosis (continuous), race, gender, marital status, education, and employment status as operationalized in Table 1. In our analyses, we operationalize race as a social construct that reflects the self‐classification of persons into racialized groups [50, 51, 52]. Within each MSA stratum, analyses were stratified by race (non‐Hispanic Black and non‐Hispanic White) and sex (female and male). Cell sizes < 11 were suppressed to protect participants' privacy. Statistical significance in 2‐sided tests was defined as α = 0.05. All analyses were performed using SAS version 9.4 (SAS Institute Inc., Cary, NC).

## 3 Results

Among 2805 cancer survivors (mean age 57.8 years), most were female (64%), racialized as non‐Hispanic White (83%), employed at enrollment (47%), and had at least a college education (47%). Breast (24%), uterine (12%), and colorectal (8%) cancers were the most common, with a mean of 3.7 years from diagnosis to cohort enrollment. Higher mortgage denial risk was concentrated among younger, female, non‐Hispanic Black participants who were less likely to be married, employed, or have higher education (Table 1).

**TABLE 1:** UNC Cancer Survivorship Cohort participant characteristics, stratified by MSA‐standardized mortgage denial risk.

	Overall (*N* = 2805)	MSA‐standardized mortgage denial risk^a^	
		Low (*N* = 1760)	High (*N* = 1045)
	*N* (%)		
Age at diagnosis, mean [SD]	57.8 [12.6]	58.3 [12.4]	57.1 [12.9]
< 55 years	1093 (39)	653 (37)	440 (42)
55–64 years	871 (31)	569 (32)	302 (29)
65+ years	841 (30)	538 (31)	303 (29)
Race			
Non‐Hispanic White	2323 (83)	1543 (88)	780 (75)
Non‐Hispanic Black	409 (15)	174 (10)	235 (23)
Non‐Hispanic another underrepresented race^b^	73 (4)	43 (2)	30 (3)
Sex			
Female	1802 (64)	1093 (62)	709 (68)
Male	1003 (36)	667 (38)	336 (32)
Education			
High school graduate or less	763 (27)	382 (22)	381 (37)
Some college or technical school	714 (26)	418 (24)	296 (28)
College graduate	733 (26)	517 (29)	216 (21)
Postgraduate or professional degree	595 (21)	443 (25)	152 (15)
Currently employed			
No	1493 (53)	882 (50)	611 (59)
Yes	1312 (47)	878 (50)	434 (42)
Current marital status			
Single, never married	252 (9)	131 (7)	121 (12)
Married or living with a partner	1888 (67)	1255 (71)	633 (61)
Separated, widowed, or divorced	665 (24)	374 (21)	291 (28)
Primary cancer site			
Breast	675 (24)	439 (25)	236 (23)
Brain and other central nervous system	29 (1)	15 (1)	14 (1)
Endocrine	43 (2)	22 (1)	21 (2)
Gastrointestinal (GI)	498 (18)	311 (18)	187 (18)
Colorectal	231 (8)	153 (9)	78 (8)
Hepatobiliary	131 (5)	81 (5)	50 (5)
Other GI^c^	136 (5)	77 (4)	59 (6)
Genitourinary (GU)	562 (20)	351 (20)	211 (20)
Bladder	105 (4)	58 (3)	47 (5)
Kidney	101 (4)	58 (3)	43 (4)
Prostate	206 (7)	145 (8)	61 (6)
Other GU^d^	150 (5)	90 (5)	60 (6)
Gynecologic	441 (16)	264 (15)	177 (17)
Ovary	92 (3)	57 (3)	35 (3)
Uterine	349 (12)	207 (12)	142 (14)
Head and neck	184 (7)	129 (7)	55 (5)
Lung	86 (3)	43 (2)	43 (4)
Lymphatic and hematologic	51 (2)	34 (2)	17 (1)
Melanoma	183 (7)	121 (7)	62 (6)
Other/unknown	53 (2)	31 (2)	22 (2)
Cancer stage			
Localized	1034 (42)	646 (42)	388 (42)
Regional	1081 (44)	673 (43)	408 (44)
Distant	368 (15)	238 (15)	130 (14)
Missing	322	203	119
Any surgery			
No	879 (31)	533 (30)	346 (33)
Yes	1926 (69)	1227 (70)	699 (67)
Any chemotherapy			
No	1800 (64)	1144 (65)	656 (63)
Yes	1005 (36)	616 (35)	389 (37)
Any radiation			
No	2005 (72)	1253 (71)	752 (72)
Yes	800 (29)	507 (29)	293 (28)
Any bone marrow or other cancer treatment			
No	2431 (87)	1521 (86)	910 (87)
Yes	374 (13)	239 (14)	135 (13)
Multiple treatment types			
No	1602 (57)	1013 (58)	589 (56)
Yes	1203 (43)	747 (42)	456 (44)

Abbreviations: CI, confidence interval; FACT‐G, Functional Assessment of Cancer Therapy – General; GI, gastrointestinal; GU, genitourinary; SD, standard deviation.

^a^ Standardized relative risks of mortgage loan denial (RR_mortgage denial_) were estimated separately for each MSA. Census tracts with RR_mortgage denial_ ≤ 1 are labeled “Low,” indicating lower‐than‐average denial risk, while tracts with RR_mortgage denial_ > 1 are labeled “High,” indicating higher‐than‐average denial risk. Values are comparable within but not across MSAs.

^b^ Another Underrepresented Race includes American Indian/Native American (*n* < 11); Asian (*n* = 25); Native Hawaiian/Pacific Islander (*n* < 11); and other race not specified (*n* = 37).

^c^ Other gastrointestinal cancers include those of the anus and anal canal, esophagus, rectosigmoid junction, retroperitoneum and peritoneum, small intestine, stomach, and other digestive organs.

^d^ Other genitourinary cancers include those of the cervix, penis, testes, ureter, vagina, vulva, other female genital organs, and other unspecified urinary organs.

The overall mean FACT‐G score was 81.7, slightly above the previously validated average of 80.9 for adults with cancer [43, 46]. HRQoL scores were worse in high mortgage loan denial risk areas compared to low mortgage loan denial risk areas (79.5 vs. 83.0). Declines in HRQoL scores were most pronounced among younger, single, and lower‐educated survivors and those with gastrointestinal, ovarian, or late‐stage cancers. Older, non‐Hispanic White race, married or partnered, higher‐educated, and employed survivors generally maintained above‐average HRQoL (Table S1).

**FIGURE 2:**
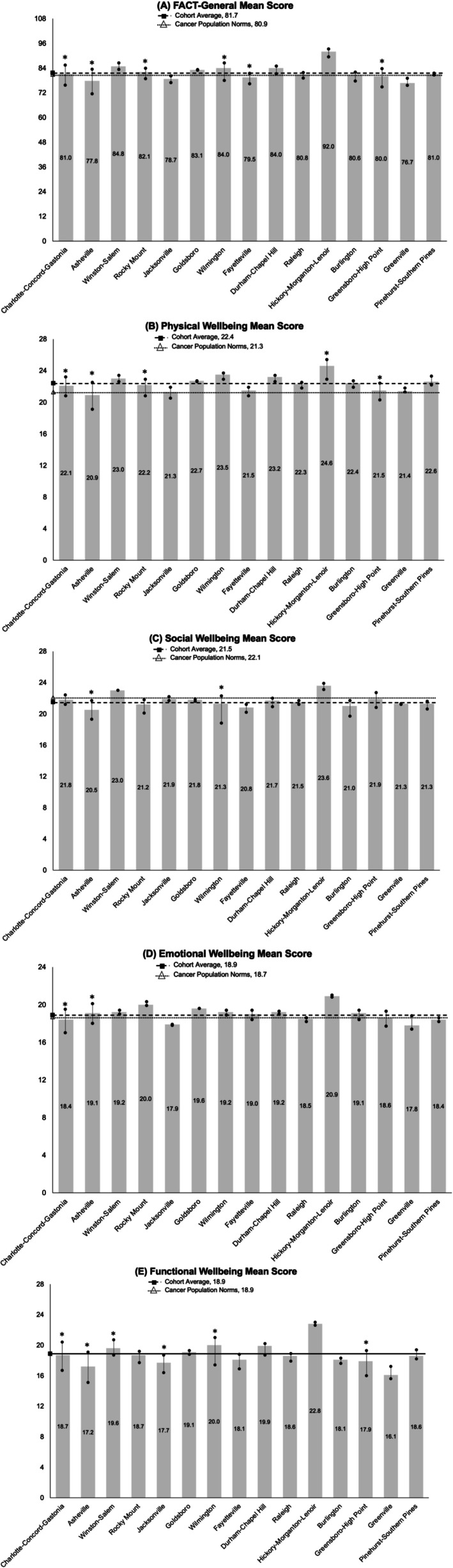
Mean overall, physical, social, emotional, and functional well‐being scores by North Carolina metropolitan statistical areas. Asterisks (*) represent a clinically meaningful difference (*p* < 0.05) between survivors at higher versus lower risk of mortgage loan denial within each North Carolina MSA. An unadjusted difference > 5 points is considered clinically meaningful for overall well‐being, while an unadjusted difference > 2 points is considered meaningful for subscale‐specific (physical, social, emotional, and functional) well‐being.

Across most North Carolina MSAs, survivors' scores across well‐being domains were generally at or below both the analytic sample mean and the previously validated average for a sample of adults with cancer (Figure 2). More than 4 in 10 survivors (42.0%) reported low overall well‐being. Nearly half reported low social (44.8%) and functional (46.6%) well‐being, while about one‐third reported low physical (32.5%) or emotional (38.3%) well‐being. In all seven MSAs, participants who resided in a higher mortgage denial risk area were more likely to report low overall well‐being, with statistically significant differences (*p* ≤ 0.05) in all MSAs except Rocky Mount and Charlotte‐Concord‐Gastonia (Table 2).

**TABLE 2:** Prevalence of low overall, physical, social, emotional, and functional well‐being by mortgage denial risk across North Carolina metropolitan statistical areas.

Mortgage denial risk	*N* ^a^	Low HRQoL		Low PWB		Low SWB		Low EWB		Low FWB	
		*n*	%	*n*	%	*n*	%	*n*	%	*n*	%
Overall	2805	1179	42.0	911	32.5	1257	44.8	1074	38.3	1307	46.6
Greensboro‐High Point											
RR_mortgage denial_ ≤ 1	106	41	38.7	37	34.9	40	37.7	42	39.6	45	42.5
RR_mortgage denial_ > 1	76	42	55.3	38	50.0	40	52.6	36	47.4	44	57.9
*χ* ^2^ *p*		0.03		0.04		0.05		0.30		0.04	
Raleigh											
RR_mortgage denial_ ≤ 1	478	192	40.2	153	32.0	209	43.7	184	38.5	222	46.4
RR_mortgage denial_ > 1	261	129	49.4	97	37.2	125	47.9	114	43.7	138	52.9
*χ* ^2^ *p*		0.02		0.16		0.28		0.17		0.09	
Durham‐Chapel Hill											
RR_mortgage denial_ ≤ 1	621	215	34.6	155	25.0	255	41.1	210	33.8	237	38.2
RR_mortgage denial_ > 1	209	101	48.3	58	27.7	103	49.3	88	42.1	107	51.2
*χ* ^2^ *p*		< 0.001		0.42		0.04		0.03		< 0.001	
Fayetteville											
RR_mortgage denial_ ≤ 1	226	93	41.2	80	35.4	104	46.0	78	34.5	109	48.2
RR_mortgage denial_ > 1	129	67	51.9	63	48.8	68	52.7	58	45.0	72	55.8
*χ* ^2^ *p*		0.05		0.01		0.22		0.05		0.17	
Wilmington											
RR_mortgage denial_ ≤ 1	58	14	24.1	14	24.1	22	37.9	23	39.7	19	32.8
RR_mortgage denial_ > 1	24	13	54.2	< 11^b^	—^b^	13	54.2	< 11^b^	—^b^	15	62.5
*χ* ^2^ *p*		0.01		0.39		0.18		0.87		0.01	
Rocky Mount											
RR_mortgage denial_ ≤ 1	63	22	34.9	13	20.6	27	42.9	16	25.4	23	36.5
RR_mortgage denial_ > 1	33	41	65.1	18	54.5	19	57.6	< 11^b^	—^b^	18	54.5
*χ* ^2^ *p*		0.12		< 0.001		0.17		0.65		0.09	
Charlotte‐Concord‐Gastonia											
RR_mortgage denial_ ≤ 1	28	11	39.3	< 11^b^	—^b^	13	46.4	11	39.3	12	42.9
RR_mortgage denial_ > 1	23	12	52.2	12	52.2	14	60.9	12	52.2	12	52.2
*χ* ^2^ *p*		0.36		0.02		0.30		0.36		0.51	

Abbreviations: EWB, emotional well‐being; FWB, functional well‐being; HRQoL, health‐related quality of life; PWB, physical well‐being; RR, relative risk; SWB, social well‐being.

^a^ Excluded participants with missing marital status (*n* = 26) and education or employment (*n* = 18).

^b^ Cell sizes < 11 were suppressed to protect participants' privacy.

In Greensboro‐High Point, survivors who resided in a higher (compared to lower) mortgage denial risk area were more likely to report low overall well‐being (55.3% vs. 38.7%; *p* = 0.03) as well as low physical, social, and functional well‐being (all *p* ≤ 0.05). Fayetteville survivors who resided in a higher (compared to lower) risk of mortgage loan denial area were more likely to report lower overall well‐being (51.9% vs. 41.2%; *p* = 0.05), along with low physical and emotional well‐being (both *p* ≤ 0.05). In Charlotte‐Concord‐Gastonia, survivors who resided in a higher (compared to lower) risk of experiencing mortgage loan denial area were more likely to report lower physical well‐being (*p* = 0.02; Table 2).

In the Research Triangle, which includes the Durham‐Chapel Hill and Raleigh MSAs, survivors who resided in a higher (compared to lower) risk mortgage loan denial area were more likely to report lower overall well‐being (Durham‐Chapel Hill: 48.3% vs. 34.6%; *p* < 0.05; Raleigh: 49.4% vs. 40.2%; *p* = 0.02). Durham‐Chapel Hill survivors who resided in a higher (compared to lower) mortgage denial risk area were also more likely to report lower social, emotional, and functional well‐being (all *p* < 0.04). Wilmington survivors who resided in a higher (compared to lower) mortgage denial risk area were more likely to report lower overall (54.2% vs. 41.2%; *p* = 0.01) and functional well‐being (62.5% vs. 32.8%; *p* = 0.01). In Rocky Mount, survivors who resided in a higher (compared to lower) mortgage loan denial risk area were more likely to report lower physical well‐being (54.5% vs. 20.6%; *p* < 0.001; Table 2).

Across all seven MSAs, residence in a higher mortgage loan denial risk area was associated with lower overall mean FACT‐G scores in unadjusted models. After adjustment, point estimates remained negative in all MSAs except for Raleigh, reaching statistical significance in Greensboro‐High Point, Fayetteville, and Charlotte‐Concord‐Gastonia (Table 3). In Greensboro‐High Point, FACT‐G scores were 6.2 (95% CI: 1.5, 10.9) points lower among survivors who resided in a higher (compared to lower) mortgage loan denial risk area. Survivors' risk of mortgage loan denial was associated with a small, yet clinically meaningful difference in FACT‐G scores overall in Fayetteville. FACT‐G scores were 4.1 (0.1, 8.1) points lower among survivors who resided in a higher (compared to lower) mortgage loan denial risk area. Survivors' risk of mortgage loan denial was associated with a large and clinically meaningful difference in FACT‐G scores overall in Charlotte‐Concord‐Gastonia. FACT‐G scores in Charlotte‐Concord‐Gastonia were 9.6 (2.5, 16.8) points lower among all survivors who resided in a higher (compared to lower) mortgage loan denial risk area (Table 3).

**TABLE 3:** Differences in overall well‐being scores associated with mortgage denial risk.

NC MSAs	Mortgage denial risk	*n* ^a^	Unadjusted		Adjusted^b^	
			FACT‐G mean score	Diff. in FACT‐G score (95% CI)	FACT‐G mean score	Diff. in FACT‐G score (95% CI)
Greensboro‐High Point (*N* = 182)	RR_mortgage denial_ ≤ 1	106	83.7	Ref	80.9	Ref
	RR_mortgage denial_ > 1	76	74.8	−8.9 (−13.5, −4.2)	74.7	−6.2 (−10.9, −1.5)
Raleigh (*N* = 739)	RR_mortgage denial_ ≤ 1	478	81.8	Ref	72.9	Ref
	RR_mortgage denial_ > 1	26	79.1	−2.7 (−5.3, −0.1)	73.4	0.5 (−2.1, 3.1)
Durham‐Chapel Hill (*N* = 830)	RR_mortgage denial_ ≤ 1	621	84.9	Ref	82.0	Ref
	RR_mortgage denial_ > 1	209	81.2	−3.8 (−6.3, −1.3)	79.9	−2.1 (−4.7, 0.4)
Fayetteville (*N* = 355)	RR_mortgage denial_ ≤ 1	226	81.4	Ref	79.7	Ref
	RR_mortgage denial_ > 1	129	76.3	−4.9 (−9.0, −0.9)	75.7	−4.1 (−8.1, −0.1)
Wilmington (*N* = 82)	RR_mortgage denial_ ≤ 1	58	86.5	Ref	74.4	Ref
	RR_mortgage denial_ > 1	24	78.0	−8.5 (−16.3, −0.7)	73.7	−0.7 (−8.8, 7.4)
Rocky Mount (*N* = 96)	RR_mortgage denial_ ≤ 1	63	83.9	Ref	85.4	Ref
	RR_mortgage denial_ > 1	33	78.8	−5.1 (−12.7, 2.5)	83.4	−2.0 (−8.9, 4.9)
Charlotte‐Concord‐Gastonia (*N* = 51)	RR_mortgage denial_ ≤ 1	28	85.4	Ref	95.2	Ref
	RR_mortgage denial_ > 1	23	75.7	−9.7 (−17.4, −2.0)	85.6	−9.6 (−16.8, −2.5)

Abbreviations: CI, confidence interval; FACT‐G, Function Assessment of Cancer Therapy – General; MSA, metropolitan statistical area; NC, North Carolina; RR, relative risk.

^a^ Excluded participants with missing marital status (*n* = 26) and education or employment (*n* = 18).

^b^ Adjusted models control for age at diagnosis, race, gender, marital status, education, and employment status.

FACT‐G score differences by MSAs across racial groups were evaluated in Raleigh, Durham‐Chapel Hill, and Fayetteville, where each mortgage denial risk group included > 11 participants in both Black and White strata. In multivariable‐adjusted results, FACT‐G scores were generally similar across mortgage denial risk within sex (Table S2) and racial groups, except for White survivors in Fayetteville, who had lower HRQoL in higher versus lower mortgage loan denial risk areas (adjusted difference: −4.8, 95% CI: −9.5, −0.1; Table 4). In multivariable‐adjusted MSA‐standardized models, FACT‐G scores were generally comparable across low and high mortgage denial risk groups, with no statistically significant differences observed overall or in sex‐stratified analyses. Among White survivors, FACT‐G scores were 1.7 (0.1, 3.3) points lower among those who resided in a higher (compared to lower) mortgage loan denial risk area (Table S3).

**TABLE 4:** Differences in overall well‐being scores associated with mortgage denial risk, stratified by race.

NC MSAs	Race and mortgage denial risk	*n* ^a^	Unadjusted		Adjusted^b^	
			FACT‐G mean score	Diff. in FACT‐G score (95% CI)	FACT‐G mean score	Diff. in FACT‐G score (95% CI)
Raleigh (*N* = 719)	White, RR_mortgage denial_ ≤ 1	440	82.5	Ref	79.1	Ref
	White, RR_mortgage denial_ > 1	205	80.5	−2.0 (−4.8, 0.83)	79.2	0.1 (−2.7, 2.9)
	Black, RR_mortgage denial_ ≤ 1	27	76.3	−6.3 (−12.9, 0.4)	70.0	−3.6 (−10.0, 2.8)
	Black, RR_mortgage denial_ > 1	47	75.6	−7.0 (−12.1, −1.83)	72.7	−1.5 (−6.7, 3.7)
Durham‐Chapel Hill (*N* = 809)	White, RR_mortgage denial_ ≤ 1	544	85.2	Ref	83.1	Ref
	White, RR_mortgage denial_ > 1	148	82.5	−2.7 (−5.6, 0.11)	81.9	−1.3 (−4.2, 1.6)
	Black, RR_mortgage denial_ ≤ 1	71	83.5	−1.7 (−5.6, 2.2)	84.0	1.5 (−2.4, 5.4)
	Black, RR_mortgage denial_ > 1	57	78.4	−6.9 (−11.1, −2.6)	79.2	−2.9 (−7.3, 1.4)
Fayetteville (*N* = 344)	White, RR_mortgage denial_ ≤ 1	180	81.2	Ref	79.1	Ref
	White, RR_mortgage denial_ > 1	79	75.5	−5.7 (−10.7, −0.7)	74.4	−4.8 (−9.5, −0.1)
	Black, RR_mortgage denial_ ≤ 1	39	83.1	1.9 (−4.6, 8.5)	80.4	3.8 (−2.5, 10.1)
	Black, RR_mortgage denial_ > 1	46	76.9	−4.3 (−10.4, 1.9)	76.0	−1.4 (−7.2, 4.5)

Abbreviations: CI, confidence interval; FACT‐G, Function Assessment of Cancer Therapy – General; MSA, metropolitan statistical area; NC, North Carolina; RR, relative risk.

^a^ Excluded participants with missing marital status (*n* = 26) and education or employment (*n* = 18).

^b^ Adjusted models control for age at diagnosis, race, gender, marital status, education, and employment status.

Physical well‐being scores differed only in Charlotte‐Concord‐Gastonia, where survivors who resided in a higher mortgage loan denial risk area scored 2.7 (0.3, 5.1) points lower than those who resided in a lower mortgage loan denial risk area (Table S4). Emotional well‐being scores were also lower among survivors who resided in a higher (compared to lower) risk area, with a 1.7‐point (0.2, 3.2) lower difference in Greensboro‐High Point and a 2.4‐point (0.3, 4.5) lower difference in Charlotte‐Concord‐Gastonia (Table S5). Differences in functional well‐being scores were 2.1 (0.2, 4.1) points lower among survivors who resided in a higher (compared to lower) mortgage loan denial risk area in Greensboro‐High Point (Table S6). No significant differences in social well‐being according to risk of mortgage denial were observed across the seven MSAs (Table S7).

## 4 Discussion

In this sample of predominantly non‐Hispanic White, highly educated cancer survivors who accessed care at an NCI‐designated Comprehensive Cancer Center, more than a third reported low overall and subscale‐specific well‐being. We observed statistically significant and clinically meaningful differences in overall well‐being among Greensboro‐High Point, Fayetteville, and Charlotte‐Concord‐Gastonia survivors. Clinically meaningful differences in physical well‐being were observed in Charlotte‐Concord‐Gastonia; functional well‐being in Greensboro‐High Point; and emotional well‐being in both Greensboro‐High Point and Charlotte‐Concord‐Gastonia.

These differences may reflect the unique historical and socioeconomic contexts of these MSAs, which likely shape both housing access and survivors' well‐being. Greensboro‐High Point, known for its textile manufacturing and its Civil Rights Movement legacy, is home to North Carolina Agricultural and Technical State University (NC A&T State), the state's first and the nation's largest Historically Black College and University (HBCU) by enrollment [53, 54]. While an HBCU may bolster community support, structural housing inequities can limit survivors' financial and health resources. Fayetteville, one of the fastest‐growing MSAs, benefits from its proximity to Fort Bragg, one of the largest military installations in the world [55], and strong national corporate investment. Military installations may provide economic stability, but residents outside these networks can face greater homeownership barriers, leading to increased stress and lower self‐rated health. Charlotte‐Concord‐Gastonia, North Carolina's largest MSA and a leading U.S. financial hub, has rapidly urbanized [56, 57, 58], potentially intensifying housing competition and lending discrimination in addition to amplifying the impact of mortgage denial on survivor well‐being. Such distinct histories informed structural inequities in housing access, wealth, and opportunities, all of which provide important context for survivors' HRQoL.

The Research Triangle, comprised of the Durham‐Chapel Hill and Raleigh MSAs, is known for its innovation‐driven economy and strong government‐academia‐industry collaboration [59] and showed modest overall HRQoL differences. However, Durham‐Chapel Hill survivors who resided in a higher mortgage denial risk area reported significantly lower well‐being scores, indicating persistent structural barriers in economically dynamic regions. In Wilmington, with a rich military and maritime history [60, 61], survivors who resided in a higher risk mortgage denial area reported lower overall and functional well‐being. Rocky Mount, shaped by agriculture and tobacco industries [62], showed that a higher risk for mortgage loan denial was associated with lower physical well‐being, though the limited sample size warrants cautious interpretation. Our findings further reflect how layered histories, regional economics, and systemic mortgage discrimination intersect to shape survivors' experiences.

Research increasingly recognizes the role of geography in shaping cancer outcomes, with most studies focusing on urban–rural differences [63, 64, 65, 66, 67]. Rural survivors often face poorer outcomes due to limited healthcare access and socioeconomic disadvantage [65]. Urban areas with seemingly greater resources also manifest health inequities [68, 69, 70, 71, 72, 73]. Urban areas are not monolithic; decades of structurally racist policies like redlining, urban renewal, exclusionary zoning, and disinvestment have created unequal conditions and access to resources, disproportionately affecting underserved communities.

Disparities among urban cancer survivors highlight how racial and socioeconomic factors shape neighborhoods within MSAs and influence cancer outcomes. The HMDA, designed to track neighborhood‐level mortgage discrimination, reveals how systemic disinvestment hinders homeownership and wealth building, instead favoring rental markets [15, 34, 35]. Such barriers amplify stress and constrain survivors' ability to manage the physical and financial burden of cancer, contributing to challenges in maintaining health‐promoting behaviors, including physical activity and moderated substance use [19, 74, 75]. High‐renter neighborhoods face greater residential instability and weaker social support networks, worsening outcomes across the cancer care continuum [76, 77].

Establishing how area‐level housing and investment patterns affect HRQoL can inform targeted interventions for survivors. Potential strategies include expanding access to affordable mortgage and rental assistance programs in high‐risk neighborhoods, leveraging local institutions (e.g., HBCUs, military support networks, and community centers) to deliver survivorship resources, and implementing community‐based programs that strengthen social cohesion and resilience. Clinically, providers can integrate financial and housing counseling into survivorship care plans and screen for neighborhood‐level risk factors affecting HRQoL. Optimizing HRQoL not only guides survivors' treatment and lifestyle choices but also serves as a valuable proxy for the broader cancer survivorship experience [78, 79, 80].

Previous studies estimated mean FACT‐G scores across diverse cancer cohorts, but comparisons are limited by varying cancer types and low well‐being cutoffs [44, 45, 81, 82, 83]. Although FACT‐G use has expanded to more diverse populations, normative data for disease‐, symptom‐, or condition‐specific subscales remain limited [43]. In the UNC Cancer Survivorship Cohort, non‐Hispanic Black survivors had a mean FACT‐G score of 78.4, slightly higher than the 76.0 reported in the Detroit Research on Cancer Survivors (ROCS) study (76.0), which includes Black survivors diagnosed with breast, colorectal, lung, and prostate cancers [44]. Detroit, a large, underserved MSA with high poverty and persistent racial segregation, faces limited access to economic, political, and social resources [84, 85]. Similarly, North Carolina MSAs reflect the enduring impact of racial segregation and growing neighborhood homogeneity, contributing to unequal access to health‐promoting sources for survivors [86, 87].

Strengths of this work include using a diverse cohort of survivors from a North Carolina Comprehensive Cancer Center in a region shaped by a history of racial injustices and manifestations of structural racism. The detailed survey instruments incorporated a validated HRQoL measure (FACT‐G) and controlled for key confounders. Inclusion of several common cancers enhanced the generalizability of our findings beyond a single cancer type. A key innovation of this work is the application of a validated, publicly available, and spatially explicit mortgage denial risk metric, offering a novel lens to examine neighborhood‐level structural housing inequities relevant to cancer survivorship.

While capturing key structure disinvestment, this measure reflects mortgage denial risk at one time point between 2010 and 2016 and HMDA data from 2010 to 2014, limiting the assessment of residential mobility and establishing temporality. HMDA data also have known limitations, including omitted‐variable bias from unmeasured factors such as borrower credit scores, loan officer discretion, and other determinants not captured within the HMDA database [88, 89]. Further, our analysis does not account for individual housing status, including renter status or denial, nor other contextual factors such as neighborhood‐level social and environmental factors underlying the observed associations. While our findings may not be generalizable beyond the Southeastern U.S., the racial diversity, geographic variation, and structural inequities within North Carolina reflect broader national patterns [17].

Our sample primarily included older female survivors at an outpatient oncology clinic, likely representing better cancer care access and potentially lower mortgage loan denial risk. This sociodemographic profile, excluding participants with multiple cancer diagnoses, may bias our findings towards healthier, more advantaged survivors, underestimating the impact of structural housing inequities on HRQoL. We further acknowledge possible residual confounding from unmeasured individual‐level factors such as household income and housing tenure, which could influence the association between mortgage loan denial risk and HRQoL. Because our data precede major sociopolitical events, including the COVID‐19 pandemic, our findings may not fully reflect contemporary housing and economic conditions.

## 5 Conclusion

Rapid growth, gentrification, and development across North Carolina may be reshaping neighborhoods, with the state projected to become the seventh most populous state in the U.S. by the early 2030s [86]. Even among a relatively advantaged group of cancer survivors in North Carolina, our study highlights that a higher neighborhood‐level risk of mortgage loan denial is associated with worse overall well‐being. Future studies should investigate longitudinal associations using updated HMDA data, housing inequity metrics, and broader socioeconomic and policy factors to clarify underlying mechanisms. Our findings can inform strategies to better support survivors and improve well‐being beyond the scope of cancer care.

## Author Contributions

**Jordyn A. Brown:** conceptualization (lead), formal analysis (lead), investigation (lead), writing – original draft (lead), writing – review and editing (lead). **Taylor D. Ellington:** conceptualization (supporting), writing – review and editing (supporting). **Leah Moubadder:** conceptualization (supporting), methodology (lead), writing – review and editing (supporting). **Maya Bliss:** conceptualization (supporting), methodology (lead), writing – review and editing (supporting). **Anjali D. Kumar:** conceptualization (supporting), writing – review and editing (supporting). **Chantel L. Martin:** conceptualization (supporting), writing – review and editing (supporting). **Laura Farnan:** resources (lead), software (lead), writing – review and editing (supporting). **Adrian Gerstel:** project administration (lead), writing – review and editing (supporting). **Lauren E. McCullough:** conceptualization (supporting), methodology (lead), writing – review and editing (supporting). **Hazel B. Nichols:** conceptualization (supporting), supervision (lead), writing – review and editing (supporting).

## Funding

The generation of the contemporary mortgage data and mortgage denial risk measure was supported in part by the National Institute of Health's National Cancer Institute (R01CA259192 for L.E.M.). This work was supported in part by the NIH National Cancer Institute Cancer Care Quality Training Program (T32CA116339 for J.A.B.) and Cancer Control Education Program (T32CA057726 for T.D.E.).

## Ethics Statement

This project was reviewed and approved by the Human Research Protections Program (IRB Number: 23–1585) at the University of North Carolina at Chapel Hill.

## Conflicts of Interest

The authors declare no conflicts of interest.

## Supporting information


**Tables S1–S7:** cam471433‐sup‐0001‐TablesS1‐S7.docx.

## Data Availability

The data generated in this study are available by request from the UNC Cancer Survivorship Cohort with Institutional Review Board approval and signed data use agreements.
