# Supplementary material for: Contemporary Area‐Level Mortgage Loan Denial Risk and Health‐Related Quality of Life Among Cancer Survivors
Source: Cancer Med. 2025 Dec 17;14(24):e71433. doi: 10.1002/cam4.71433 (PMC12710087; doi:10.1002/cam4.71433)
Supplement: Supplementary file 1 — Tables S1–S7: cam471433‐sup‐0001‐TablesS1‐S7.docx. [file CAM4-14-e71433-s001.docx]

**Supplemental Table 1**. UNC Cancer Survivorship Cohort participant characteristics, stratified by MSA-standardized mortgage denial risk and mean FACT-General scores

|  | **MSA-Standardized Mortgage Denial Risk**^†^ | | |
| --- | --- | --- | --- |
|  | Overall (N=2,805) | Low  (N=1,760) | High  (N=1,045) |
|  | Mean Score (95% CI) | | |
| **All Survivors** | 81.7 (81.0, 82.3) | 83.0 (82.2, 83.8) | 79.5 (78.5, 80.6) |
| **Age at Diagnosis** |  |  |  |
| <55 years | 79.5 (78.5, 80.5) | 81.5 (80.2, 82.7) | 76.6 (74.9, 78.2) |
| 55-64 years | 81.5 (80.3, 82.6) | 82.6 (81.3, 84.0) | 79.4 (77.3, 81.4) |
| 65+ years | 84.8 (83.6, 85.9) | 85.2 (83.8, 86.6) | 84.0 (82.0, 86.0) |
| **Race** |  |  |  |
| Non-Hispanic White | 82.5 (81.8, 83.2) | 83.5 (82.7, 84.3) | 80.4 (79.2, 81.7) |
| Non-Hispanic Black | 78.4 (76.7, 80.0) | 80.3 (77.9, 82.8) | 77.0 (74.6, 79.3) |
| Non-Hispanic Another Underrepresented Race^‡^ | 74.6 (70.7, 78.6) | 73.9 (69.0, 78.8) | 75.7 (69.3, 82.2) |
| **Sex** |  |  |  |
| Female | 81.1 (80.3, 81.9) | 82.5 (81.5, 83.5) | 78.9 (77.6, 80.3) |
| Male | 82.8 (81.7, 83.8) | 83.8 (82.5, 85.0) | 80.8 (78.8, 82.7) |
| **Education** |  |  |  |
| High School Graduate or Less | 77.8 (76.6, 79.0) | 79.5 (77.9, 81.2) | 76.1 (74.3, 77.9) |
| Some College or Technical School | 80.9 (79.6, 82.1) | 81.7 (80.2, 83.3) | 79.6 (77.6, 81.7) |
| College Graduate (Bachelors) | 83.6 (82.3, 84.8) | 84.6 (83.2, 86.0) | 81.1 (78.7, 83.4) |
| Postgraduate or Professional Degree | 85.3 (83.9, 86.7) | 85.2 (83.7, 86.7) | 85.6 (82.7, 88.4) |
| **Currently Employed** |  |  |  |
| No | 79.4 (78.5, 80.2) | 80.9 (79.8, 82.0) | 77.2 (75.7, 78.6) |
| Yes | 84.3 (83.4, 85.2) | 85.0 (83.9, 86.1) | 82.9 (81.2, 84.5) |
| **Current Marital Status** |  |  |  |
| Single, Never Married | 76.6 (74.5, 78.7) | 79.0 (76.2, 81.8) | 74.0 (70.8, 77.2) |
| Married or Living with a Partner | 83.4 (82.6, 84.2) | 84.4 (83.5, 85.3) | 81.5 (80.1, 82.9) |
| Separated, Widowed, or Divorced | 78.7 (77.4, 80.0) | 79.7 (78.1, 81.4) | 77.5 (75.4, 79.5) |
| **Primary Cancer Site** |  |  |  |
| Breast | 83.4 (82.1, 84.7) | 85.1 (83.5, 86.6) | 80.4 (78.1, 82.7) |
| Brain and Other Central Nervous System | 82.6 (76.3, 88.8) | 82.1 (73.8, 90.4) | 83.0 (73.6, 92.5) |
| Endocrine | 80.1 (75.0, 85.2) | 79.0 (72.2, 85.8) | 81.3 (73.6, 89.0) |
| Gastrointestinal (GI) | 79.4 (77.9, 80.9) | 80.9 (79.0, 82.7) | 76.8 (74.3, 79.4) |
| Colorectal | 80.9 (78.7, 83.1) | 82.7 (80.2, 85.3) | 77.3 (73.3, 81.3) |
| Hepatobiliary | 78.1 (75.1, 81.0) | 78.6 (75.0, 82.1) | 77.2 (72.3, 82.2) |
| Other GI^§^ | 78.0 (75.1, 80.8) | 79.5 (75.9, 83.2) | 75.9 (71.4, 80.5) |
| Genitourinary (GU) | 82.1 (80.7, 83.5) | 83.6 (81.9, 85.4) | 79.6 (77.1, 82.0) |
| Bladder | 81.0 (77.7, 84.2) | 82.5 (78.3, 86.7) | 79.1 (74.0, 84.2) |
| Kidney | 81.3 (78.0, 84.6) | 83.7 (79.5, 87.9) | 78.1 (72.7, 83.4) |
| Prostate | 85.7 (83.3, 88.0) | 86.3 (83.7, 89.0) | 84.1 (79.6, 88.6) |
| Other GU^¶^ | 78.6 (75.9, 81.3) | 80.0 (76.6, 83.4) | 76.5 (72.0, 81.0) |
| Gynecologic | 80.0 (78.4, 81.6) | 79.9 (77.9, 81.9) | 80.0 (77.4, 82.7) |
| Ovary | 75.6 (72.1, 79.0) | 78.0 (73.7, 82.2) | 71.6 (65.7, 77.5) |
| Uterine | 81.1 (79.3, 82.9) | 80.4 (78.2, 82.6) | 82.1 (79.2, 85.1) |
| Head and Neck | 82.7 (80.2, 85.2) | 83.6 (80.7, 86.4) | 80.6 (75.9, 85.4) |
| Lung | 80.9 (77.3, 84.6) | 84.3 (79.5, 89.2) | 77.5 (72.1, 82.9) |
| Lymphatic and Hematologic | 81.3 (76.6, 86.0) | 85.7 (80.2, 91.2) | 72.4 (63.9, 81.0) |
| Melanoma | 84.9 (82.5, 87.4) | 85.4 (82.5, 88.3) | 84.1 (79.6, 88.6) |
| Other/Unknown | 79.0 (74.4, 83.6) | 79.4 (73.6, 85.2) | 78.4 (70.8, 85.9) |
| **Cancer Stage** |  |  |  |
| Localized | 83.4 (82.3, 84.4) | 85.1 (83.8, 86.3) | 80.6 (78.8, 82.4) |
| Regional | 81.1 (80.1, 82.1) | 82.2 (81.0, 83.4) | 79.3 (77.5, 81.0) |
| Distant | 78.5 (76.7, 80.2) | 79.8 (77.7, 81.8) | 76.1 (73.0, 79.2) |
| **Any Surgery** |  |  |  |
| No | 80.8 (79.7, 81.9) | 82.3 (80.9, 83.7) | 78.6 (76.6, 80.5) |
| Yes | 82.1 (81.3, 82.9) | 83.3 (82.4, 84.2) | 80.0 (78.7, 81.3) |
| **Any Chemotherapy** |  |  |  |
| No | 82.6 (81.8, 83.4) | 83.8 (82.9, 84.8) | 80.5 (79.2, 81.9) |
| Yes | 80.0 (78.9, 81.1) | 81.4 (80.1, 82.7) | 77.8 (76.0, 79.6) |
| **Any Radiation** |  |  |  |
| No | 81.4 (80.6, 82.1) | 82.8 (81.9, 83.7) | 79.0 (77.7, 80.3) |
| Yes | 82.5 (81.3, 83.7) | 83.4 (82.0, 84.8) | 80.9 (78.8, 82.9) |
| **Any Bone Marrow or Other Cancer Treatment** |  |  |  |
| No | 81.3 (80.6, 81.9) | 82.6 (81.8, 83.4) | 79.0 (77.9, 80.2) |
| Yes | 84.5 (82.7, 86.2) | 85.4 (83.3, 87.5) | 82.8 (79.7, 85.8) |
| **Multiple Treatment Types** |  |  |  |
| No | 81.5 (80.6, 82.3) | 83.0 (82.0, 84.0) | 78.9 (77.5, 80.4) |
| Yes | 81.9 (81.0, 82.9) | 82.9 (81.8, 84.1) | 80.3 (78.6, 82.0) |

Abbreviations: CI, confidence interval; FACT-G, Functional Assessment of Cancer Therapy – General; GI, gastrointestinal; GU, genitourinary; SD, standard deviation

^†^Standardized relative risks of mortgage loan denial (RR_mortgage denial_) were estimated separately for each MSA. Census tracts with RRmortgage _denial_ < 1 are labeled “Low,” indicating lower-than-average denial risk, while tracts with RR_mortgage denial_ >1 are labeled “High,” indicating higher-than-average denial risk. Values are comparable within, but not across, MSAs.

^‡^Another Underrepresented Race includes American Indian/Native American (n<11); Asian (n=25); Native Hawaiian/Pacific Islander (n<11); and other race not specified (n=37)

^§^Other gastrointestinal cancers include those of the anus and anal canal, esophagus, rectosigmoid junction, retroperitoneum and peritoneum, small intestine, stomach, and other digestive organs

^¶^Other genitourinary cancers include those of the cervix, penis, testes, ureter, vagina, vulva, other female genital organs, and other unspecified urinary organs

**Supplemental Table 2**. Differences in overall well-being scores associated with mortgage denial risk, stratified by sex

| **NC MSAs** |  |  | |  | **Unadjusted** | |  | | **Adjusted**^‡^ | | |  |
| --- | --- | --- | --- | --- | --- | --- | --- | --- | --- | --- | --- | --- |
|  | **Sex** | **Mortgage**  **Denial Risk** | **n**^†^ |  | **FACT-G Mean Score** | **Diff. in FACT-G Score**  **(95% CI)** | |  | | **FACT-G**  **Mean Score** | **Diff. in FACT-G Score**  **(95% CI)** | |
| **Greensboro-High Point**  (N=182) | Female | RR_mortgage denial_ < 1 | 68 |  | 82.3 | Ref | |  | | 79.5 | Ref | |
|  |  | RR_mortgage denial_ > 1 | 55 |  | 74.6 | -7.7 (-13.5, -1.9) | |  | | 73.7 | -5.8 (-11.7, 0.1) | |
|  | Male | RR_mortgage denial_ < 1 | 38 |  | 86.2 | Ref | |  | | 81.9 | Ref | |
|  |  | RR_mortgage denial_ > 1 | 21 |  | 75.5 | -10.7 (-18.3, -3.1) | |  | | 75.1 | -6.8 (-14.7, 1.1) | |
| **Raleigh**  (N=739) | Female | RR_mortgage denial_ < 1 | 292 |  | 81.3 | Ref | |  | | 73.1 | Ref | |
|  |  | RR_mortgage denial_ > 1 | 189 |  | 78.7 | -2.6 (-5.8, 0.7) | |  | | 73.1 | 0.0 (-3.3, 3.3) | |
|  | Male | RR_mortgage denial_ < 1 | 186 |  | 82.5 | Ref | |  | | 72.1 | Ref | |
|  |  | RR_mortgage denial_ > 1 | 72 |  | 80.0 | -2.6 (-7.2, 2.1) | |  | | 73.6 | 1.4 (-3.0, 5.8) | |
| **Durham-Chapel Hill**  (N=830) | Female | RR_mortgage denial_ < 1 | 428 |  | 84.4 | Ref | |  | | 81.9 | Ref | |
|  |  | RR_mortgage denial_ > 1 | 138 |  | 80.6 | -3.8 (-6.9, -0.7) | |  | | 79.9 | -2.0 (-5.1, 1.2) | |
|  | Male | RR_mortgage denial_ < 1 | 193 |  | 86.1 | Ref | |  | | 80.0 | Ref | |
|  |  | RR_mortgage denial_ > 1 | 71 |  | 82.2 | -3.9 (-8.0, 0.2) | |  | | 77.4 | -2.6 (-7.0, 1.8) | |
| **Fayetteville**  (N=355) | Female | RR_mortgage denial_ < 1 | 132 |  | 80.9 | Ref | |  | | 80.2 | Ref | |
|  |  | RR_mortgage denial_ > 1 | 84 |  | 76.3 | -4.6 (-9.8, 0.6) | |  | | 76.5 | -3.8 (-8.9, 1.3) | |
|  | Male | RR_mortgage denial_ < 1 | 94 |  | 81.8 | Ref | |  | | 85.2 | Ref | |
|  |  | RR_mortgage denial_ > 1 | 45 |  | 76.4 | -5.4 (-12.0, 1.2) | |  | | 79.7 | -5.5 (-11.9, 0.9) | |
| **Wilmington**  (N=82) | Female | RR_mortgage denial_ < 1 | 22 |  | 86.2 | Ref | |  | | 88.5 | Ref | |
|  |  | RR_mortgage denial_ > 1 | 11 |  | 73.2 | -13.1 (-26.5, 0.3) | |  | | 74.9 | -13.6 (-27.8, 0.6) | |
|  | Male | RR_mortgage denial_ < 1 | 36 |  | 86.6 | Ref | |  | | 65.3 | Ref | |
|  |  | RR_mortgage denial_ > 1 | 13 |  | 82.1 | -4.6 (-13.8, 4.7) | |  | | 69.9 | 4.6 (-3.8, 13.0) | |
| **Rocky Mount**  (N=96) | Female | RR_mortgage denial_ < 1 | 44 |  | 84.3 | Ref | |  | | 85.2 | Ref | |
|  |  | RR_mortgage denial_ > 1 | 22 |  | 77.1 | -7.2 (-16.1, 1.6) | |  | | 82.7 | -2.4 (-10.7, 5.9) | |
|  | Male | RR_mortgage denial_ < 1 | 19 |  | 82.8 | Ref | |  | | 77.5 | Ref | |
|  |  | RR_mortgage denial_ > 1 | 11 |  | 82.2 | -0.6 (-15.0, 13.7) | |  | | 76.0 | -1.5 (-16.0, 12.9) | |

Abbreviations: CI, confidence interval; FACT-G, Function Assessment of Cancer Therapy – General, MSA, metropolitan statistical area; NC, North Carolina; RR, relative risk

^†^Excluded participants with missing marital status (n=26), education and employment (n=18)

^‡^Adjusted models control for age at diagnosis, race, gender, marital status, education, and employment status

**Supplemental Table 3**. Associations between MSA-standardized tertiles of mortgage denial risk and FACT-General scores

|  |  |  |  | **Unadjusted** | |  | **Adjusted**^§^ | |
| --- | --- | --- | --- | --- | --- | --- | --- | --- |
|  | Mortgage Denial Risk^†^ | N^‡^ |  | FACT-G Mean Score | Diff. in FACT-G Score  (95% CI) |  | FACT-G Mean Score | Diff. in FACT-G Score  (95% CI) ^‡^ |
| **Overall** |  |  |  |  |  |  |  |  |
| All Survivors | Low | 1,760 |  | 82.7 | Ref |  | 78.9 | Ref |
|  | High | 1,045 |  | 79.4 | -3.3 (-4.8, -1.9) |  | 77.4 | -1.5 (-3.1, 0.1) |
| **Race** |  |  |  |  |  |  |  |  |
| White | Low | 1,543 |  | 83.2 | Ref |  | 81.5 | Ref |
|  | High | 780 |  | 80.3 | -3.0 (-4.5, -1.4) |  | 79.8 | -1.7 (-3.3, -0.1) |
| Black | Low | 174 |  | 79.6 | Ref |  | 79.3 | Ref |
|  | High | 235 |  | 76.5 | -3.2 (-7.3, 0.9) |  | 77.6 | -1.7 (-5.8, 2.3) |
| **Sex** |  |  |  |  |  |  |  |  |
| Female | Low | 1,093 |  | 82.0 | Ref |  | 79.4 | Ref |
|  | High | 709 |  | 78.6 | -3.4 (-5.2, -1.6) |  | 77.6 | -1.9 (-3.8, 0.0) |
| Male | Low | 667 |  | 83.8 | Ref |  | 77.4 | Ref |
|  | High | 336 |  | 80.7 | -3.0 (-5.4, -0.6) |  | 76.9 | -0.5 (-2.9, 1.8) |

^†^Standardized relative risks of mortgage loan denial (RR_mortgage denial_) were estimated separately for each MSA. Census tracts with RRmortgage _denial_ < 1 are labeled “Low,” indicating lower-than-average denial risk, while tracts with RR_mortgage denial_ >1 are labeled “High,” indicating higher-than-average denial risk. Values are comparable within, but not across, MSAs.

^‡^Excluded participants with missing marital status (n=26), education and employment (n=18)

^§^Adjusted models control for age at diagnosis, race, gender, marital status, education, and employment status

**Supplemental Table 4.** Differences in physical well-being scores associated with mortgage denial risk

| **NC MSA** |  |  |  | **Unadjusted** | |  | **Adjusted**^‡^ | |
| --- | --- | --- | --- | --- | --- | --- | --- | --- |
|  | **Mortgage**  **Denial Risk** | **n**^†^ |  | **PWB**  **Mean Score** | **Diff. in PWB Score**  **(95% CI)** |  | **PWB**  **Mean Score** | **Diff. in PWB Score**  **(95% CI)** |
| **Greensboro-High Point**  (N=182) | RR_mortgage denial_ < 1 | 106 |  | 22.4 | Ref |  | 22.7 | Ref |
|  | RR_mortgage denial_ > 1 | 76 |  | 20.3 | -2.0 (-3.7, -0.4) |  | 21.4 | -1.3 (-3.0, 0.3) |
| **Raleigh**  (N=739) | RR_mortgage denial_ < 1 | 478 |  | 22.5 | Ref |  | 20.2 | Ref |
|  | RR_mortgage denial_ > 1 | 26 |  | 21.8 | -0.7 (-1.5, 0.2) |  | 20.4 | 0.1 (-0.7, 1.0) |
| **Durham-Chapel Hill**  (N=830) | RR_mortgage denial_ < 1 | 621 |  | 23.4 | Ref |  | 23.1 | Ref |
|  | RR_mortgage denial_ > 1 | 209 |  | 22.6 | -0.8 (-1.6, -0.1) |  | 22.6 | -0.5 (-1.3, 0.3) |
| **Fayetteville**  (N=355) | RR_mortgage denial_ < 1 | 226 |  | 21.9 | Ref |  | 22.7 | Ref |
|  | RR_mortgage denial_ > 1 | 129 |  | 20.8 | -1.0 (-2.2, 0.1) |  | 21.8 | -0.8 (-2.0, 0.3) |
| **Wilmington**  (N=82) | RR_mortgage denial_ < 1 | 58 |  | 23.7 | Ref |  | 22.9 | Ref |
|  | RR_mortgage denial_ > 1 | 24 |  | 22.9 | -0.8 (-3.1, 1.4) |  | 23.3 | 0.4 (-2.1, 2.8) |
| **Rocky Mount**  (N=96) | RR_mortgage denial_ < 1 | 63 |  | 22.9 | Ref |  | 24.0 | Ref |
|  | RR_mortgage denial_ > 1 | 33 |  | 20.8 | -2.2 (-4.6, 0.3) |  | 22.8 | -1.2 (-3.5, 1.0) |
| **Charlotte-Concord-Gastonia**  (N=51) | RR_mortgage denial_ < 1 | 28 |  | 23.2 | Ref |  | 26.3 | Ref |
|  | RR_mortgage denial_ > 1 | 23 |  | 20.8 | -2.4 (-5.0, 0.2) |  | 23.6 | -2.7 (-5.1, -0.3) |

Abbreviations: CI, confidence interval; MSA, metropolitan statistical area; NC, North Carolina; NE, not estimable; PWB; physical well-being; RR, relative risk

^†^Excluded participants with missing marital status (n=26), education and employment (n=18)

^‡^Adjusted models control for age at diagnosis, race, gender, marital status, education, and employment status

**Supplemental Table 5.** Differences in emotional well-being scores associated with mortgage denial risk

| **NC MSA** |  | |  | **Unadjusted** | |  | **Adjusted**^‡^ | |
| --- | --- | --- | --- | --- | --- | --- | --- | --- |
|  | **Mortgage**  **Denial Risk** | **n**^†^ |  | **EWB Mean Score** | **Diff. in EWB Score**  **(95% CI)** |  | **EWB**  **Mean Score** | **Diff. in EWB Score**  **(95% CI)** |
| **Greensboro-High Point**  (N=182) | RR_mortgage denial_ < 1 | 106 |  | 19.3 | Ref |  | 19.4 | Ref |
|  | RR_mortgage denial_ > 1 | 76 |  | 17.7 | -1.6 (-3.0, -0.1) |  | 17.7 | -1.7 (-3.2, -0.2) |
| **Raleigh**  (N=739) | RR_mortgage denial_ < 1 | 478 |  | 18.6 | Ref |  | 18.3 | Ref |
|  | RR_mortgage denial_ > 1 | 26 |  | 18.2 | -0.4 (-1.2, 0.4) |  | 18.2 | 0.0 (-0.8, 0.8) |
| **Durham-Chapel Hill**  (N=830) | RR_mortgage denial_ < 1 | 621 |  | 19.3 | Ref |  | 20.0 | Ref |
|  | RR_mortgage denial_ > 1 | 209 |  | 19.0 | -0.3 (-1.1, 0.5) |  | 19.5 | -0.5 (-1.3, 0.3) |
| **Fayetteville**  (N=355) | RR_mortgage denial_ < 1 | 226 |  | 19.4 | Ref |  | 20.1 | Ref |
|  | RR_mortgage denial_ > 1 | 129 |  | 18.4 | -1.0 (-2.1, 0.1) |  | 19.1 | -1.1 (-2.2, 0) |
| **Wilmington**  (N=82) | RR_mortgage denial_ < 1 | 58 |  | 19.4 | Ref |  | 17.9 | Ref |
|  | RR_mortgage denial_ > 1 | 24 |  | 18.9 | -0.5 (-3.0, 2.0) |  | 18.2 | 0.3 (-2.4, 3.0) |
| **Rocky Mount**  (N=96) | RR_mortgage denial_ < 1 | 63 |  | 19.9 | Ref |  | 20.3 | Ref |
|  | RR_mortgage denial_ > 1 | 33 |  | 20.3 | 0.3 (-1.7, 2.4) |  | 21.2 | 0.9 (-1.0, 2.9) |
| **Charlotte-Concord-Gastonia**  (N=51) | RR_mortgage denial_ < 1 | 28 |  | 19.5 | Ref |  | 23.2 | Ref |
|  | RR_mortgage denial_ > 1 | 23 |  | 17.0 | -2.5 (-5.0, 0.1) |  | 20.8 | -2.4 (-4.5, -0.3) |

Abbreviations: CI, confidence interval; EWB, emotional well-being; MSA, metropolitan statistical area; NC, North Carolina; RR, relative risk

^†^Excluded participants with missing marital status (n=26), education and employment (n=18)

^‡^Adjusted models control for age at diagnosis, race, gender, marital status, education, and employment status

**Supplemental Table 6**. Differences in functional well-being scores associated with mortgage denial risk

| **NC MSA** |  | |  | **Unadjusted** | |  | **Adjusted**^‡^ | |
| --- | --- | --- | --- | --- | --- | --- | --- | --- |
|  | **Mortgage**  **Denial Risk** | **n**^†^ |  | **FWB Mean Score** | **Diff. in FWB Score**  **(95% CI)** |  | **FWB**  **Mean Score** | **Diff. in FWB Score**  **(95% CI)** |
| **Greensboro-High Point**  (N=182) | RR_mortgage denial_ < 1 | 106 |  | 19.3 | Ref |  | 18.2 | Ref |
|  | RR_mortgage denial_ > 1 | 76 |  | 16.0 | -3.3 (-5.3, -1.3) |  | 16.1 | -2.1 (-4.1, -0.2) |
| **Raleigh**  (N=739) | RR_mortgage denial_ < 1 | 478 |  | 18.9 | Ref |  | 16.3 | Ref |
|  | RR_mortgage denial_ > 1 | 26 |  | 17.9 | -1.0 (-2.1, 0.0) |  | 16.0 | 0.3 (-0.8, 1.4) |
| **Durham-Chapel Hill**  (N=830) | RR_mortgage denial_ < 1 | 621 |  | 20.2 | Ref |  | 18.9 | Ref |
|  | RR_mortgage denial_ > 1 | 209 |  | 18.7 | -1.5 (-2.6, -0.5) |  | 18.2 | -0.7 (-1.8, 0.3) |
| **Fayetteville**  (N=355) | RR_mortgage denial_ < 1 | 226 |  | 18.8 | Ref |  | 18.1 | Ref |
|  | RR_mortgage denial_ > 1 | 129 |  | 16.9 | -1.9 (-3.5, -0.3) |  | 16.7 | -1.4 (-3.0, 0.2) |
| **Wilmington**  (N=82) | RR_mortgage denial_ < 1 | 58 |  | 21.0 | Ref |  | 16.7 | Ref |
|  | RR_mortgage denial_ > 1 | 24 |  | 17.4 | -3.7 (-6.7, -0.6) |  | 16.0 | -0.7 (-3.9, 2.5) |
| **Rocky Mount**  (N=96) | RR_mortgage denial_ < 1 | 63 |  | 19.2 | Ref |  | 20.8 | Ref |
|  | RR_mortgage denial_ > 1 | 33 |  | 17.7 | -1.5 (-4.6, 1.5) |  | 20.3 | -0.5 (-3.3, 2.3) |
| **Charlotte-Concord-Gastonia**  (N=51) | RR_mortgage denial_ < 1 | 28 |  | 20.4 | Ref |  | 24.9 | Ref |
|  | RR_mortgage denial_ > 1 | 23 |  | 16.7 | -3.7 (-7.3, 0.0) |  | 21.4 | -3.4 (-6.9, 0.0) |

Abbreviations: CI, confidence interval; FWB, functional well-being; MSA, metropolitan statistical area; NC, North Carolina; RR, relative risk

^†^Excluded participants with missing marital status (n=26) and education or employment (n=18)

^‡^Adjusted models control for age at diagnosis, race, gender, marital status, education, and employment status

**Supplemental Table 7.** Differences in social well-being scores associated with mortgage denial risk

| **NC MSA**^†^ |  | | |  | | **Unadjusted** | | |  | | **Adjusted**^‡^ | | |
| --- | --- | --- | --- | --- | --- | --- | --- | --- | --- | --- | --- | --- | --- |
|  | **Mortgage**  **Denial Risk** | **n** |  | | **SWB Mean Score** | | **Diff. in SWB Score**  **(95% CI)** |  | | **SWB**  **Mean Score** | | **Diff. in SWB Score**  **(95% CI)** |  |
| **Greensboro-High Point**  (N=182) | RR_mortgage denial_ < 1 | 106 |  | | 22.7 | | Ref |  | | 20.6 | | Ref |  |
|  | RR_mortgage denial_ > 1 | 76 |  | | 20.8 | | -1.9 (-3.4, -0.4) |  | | 19.5 | | -1.1 (-2.6, 0.5) |  |
| **Raleigh**  (N=739) | RR_mortgage denial_ < 1 | 478 |  | | 21.7 | | Ref |  | | 18.5 | | Ref |  |
|  | RR_mortgage denial_ > 1 | 26 |  | | 21.2 | | -0.5 (-1.4, 0.3) |  | | 18.4 | | 0.1 (-0.7, 0.9) |  |
| **Durham-Chapel Hill**  (N=830) | RR_mortgage denial_ < 1 | 621 |  | | 22.0 | | Ref |  | | 20.0 | | Ref |  |
|  | RR_mortgage denial_ > 1 | 209 |  | | 20.9 | | -1.1 (-2.0, -0.2) |  | | 19.7 | | -0.4 (-1.3, 0.5) |  |
| **Fayetteville**  (N=355) | RR_mortgage denial_ < 1 | 226 |  | | 21.2 | | Ref |  | | 18.8 | | Ref |  |
|  | RR_mortgage denial_ > 1 | 129 |  | | 20.2 | | -1.1 (-2.4, 0.3) |  | | 18.1 | | -0.7 (-2.0, 0.6) |  |
| **Wilmington**  (N=82) | RR_mortgage denial_ < 1 | 58 |  | | 22.3 | | Ref |  | | 17.0 | | Ref |  |
|  | RR_mortgage denial_ > 1 | 24 |  | | 18.8 | | -3.5 (-6.3, -0.8) |  | | 16.2 | | -0.7 (-3.5, 2.1) |  |
| **Rocky Mount**  (N=96) | RR_mortgage denial_ < 1 | 63 |  | | 21.8 | | Ref |  | | 20.3 | | Ref |  |
|  | RR_mortgage denial_ > 1 | 33 |  | | 20.1 | | -1.7 (-4.0, 0.6) |  | | 19.1 | | -1.2 (-3.3, 1.0) |  |
| **Charlotte-Concord-Gastonia**  (N=51) | RR_mortgage denial_ < 1 | 28 |  | | 22.4 | | Ref |  | | 20.8 | | Ref |  |
|  | RR_mortgage denial_ > 1 | 23 |  | | 21.2 | | -1.2 (-3.7, 1.3) |  | | 19.7 | | -1.1 (-3.7, 1.6) |  |

Abbreviations: CI, confidence interval; MSA, metropolitan statistical area; NC, North Carolina; RR, relative risk; SWB, social well-being

^†^Excluded participants with missing marital status (n=26) and education or employment (n=18)

^‡^Adjusted models control for age at diagnosis, race, gender, marital status, education, and employment status
